# Joint latent class model: Simulation study of model properties and application to amyotrophic lateral sclerosis disease

**DOI:** 10.1186/s12874-021-01377-9

**Published:** 2021-09-30

**Authors:** Maéva Kyheng, Génia Babykina, Camille Ternynck, David Devos, Julien Labreuche, Alain Duhamel

**Affiliations:** 1 ULR 2694 - METRICS : évaluation des technologies de santé et des pratiques médicales, Univ. Lille, CHU Lille, Lille, France; 2 Département de Biostatistiques, CHU Lille, Lille, France; 3 Expert center for ALS, Expert center for Parkinson, Medical Pharmacology, Univ. Lille, Lille Neuroscience & Cognition, Inserm, UMR-S1172, Lille, France

**Keywords:** Joint model, Latent classes, Survival analysis, Linear mixed model, MLE properties, Monte Carlo simulations, Amyotrophic lateral sclerosis

## Abstract

**Background:**

In many clinical applications, evolution of a longitudinal marker is censored by an event occurrence, and, symmetrically, event occurrence can be influenced by the longitudinal marker evolution. In such frameworks joint modeling is of high interest. The Joint Latent Class Model (JLCM) allows to stratify the population into groups (classes) of patients that are homogeneous both with respect to the evolution of a longitudinal marker and to the occurrence of an event; this model is widely employed in real-life applications. However, the finite sample-size properties of this model remain poorly explored.

**Methods:**

In the present paper, a simulation study is carried out to assess the impact of the number of individuals, of the censoring rate and of the degree of class separation on the finite sample size properties of the JLCM. A real-life application from the neurology domain is also presented. This study assesses the precision of class membership prediction and the impact of covariates omission on the model parameter estimates.

**Results:**

Simulation study reveals some departures from normality of the model for survival sub-model parameters. The censoring rate and the number of individuals impact the relative bias of parameters, especially when the classes are weakly distinguished. In real-data application the observed heterogeneity on individual profiles in terms of a longitudinal marker evolution and of the event occurrence remains after adjusting to clinically relevant and available covariates;

**Conclusion:**

The JLCM properties have been evaluated. We have illustrated the discovery in practice and highlights the usefulness of the joint models with latent classes in this kind of data even with pre-specified factors. We made some recommendations for the use of this model and for future research.

## Background

Joint models for longitudinal and time-to-event data are now widespread due to large cohort studies allowing collection of repeated measures of biomarkers and clinical events times [1]. The most popular way to analyze this kind of combined data are the *shared random effects models*, proposed by Wulfsohn and Tsiatis [2], where a function of random effects, issued from the model for longitudinal marker, is included as a covariate into the survival model. This approach allows to explain the relation between a longitudinal parameter and a time-to-event, assuming a homogeneous population. However, for certain diseases, the homogeneity assumption is not met and existence of different profiles of biomarker progression and/or of the time to-event should be accounted for in the model.

Mixture models are widely used in medical research. Different extensions allowing to account for the potential heterogeneity in population were proposed. Verbeke and Lesaffre [3] extended the mixture model to longitudinal data, assuming a latent profile of the biomarker progression (growth mixture model GMM). Muthén and Shedden [4] jointly studied longitudinal data with a binary outcome. Lin et al. [5] developed the joint latent class model (JLCM) replacing the binary outcome by a time-to-event. The JLCM allows firstly to account for the dependency between a longitudinal biomarker and a time-to-event by distinguishing between different profiles of biomarker progression associated with the risk of event. Secondly, it allows to analyze different profiles of longitudinal biomarker process censored by the event occurrence. Finally, the JLCM provides predictions for the risk of event conditional on the biomarker progression.

Very flexible, the JLCM remains quite complex. Indeed, it is composed of 3 sub-models (a multinomial logistic regression for latent classes, a linear mixed model for longitudinal process and a survival model for the time-to-event) and each of these sub-models can include covariates with effects specific or common to the latent classes.

To our knowledge, very few papers deal with studying the properties and the behaviour of the JLCM, for example Proust-Lima et al. [6], therefore it is rarely used in published clinical studies. Using a literature search of MEDLINE and WOS until december 2020, we found only 8 medical papers published since the model development in 2002 [5]. These papers appeared following a comprehensive methodology review concerning the JLCM [7] and have different objectives. These objectives can be summarized as follows: 1) to study the relationship between a longitudinal biomarker and the risk of event [8–11]; 2) to identify sub-groups of longitudinal biomarker progression censored by the event occurrence [12]; 3) to study the impact of different factors on the longitudinal biomarker progression censored by the event occurrence [13]; 4) to predict the risk of an event based on the longitudinal biomarker progression [14, 15]. Different implementations of the model were proposed to achieve a same objective. For example, for the first objective, Syrjälä et al. [8] search for the relation between childhood food consumption and the risk of advanced islet autoimmunity using a JLCM without covariates; Brilleman et al. [9] explore the relationship between the changes in body mass index and the risk of death and/or transplant in hemodialysis patients by means of the JLCM for competing risks, including the pre-specified covariates with a common effect on latent classes only in the survival sub-model; Ogata et al. [10] and Portegies et al. [11] analyze the association between fasting plasma glucose progression and the risk of cardiovascular disease and the association between the blood pressure trajectories and the risk of stroke respectively by including the pre-specified covariates with a latent class-specific effect into the linear mixed sub-model and into the survival sub-model. As other examples, for the fourth objective, [14] search to prevent Alzeihmer disease using MMSE (*Mini-Mental State Examination*) score progression and creating a predictive risk model with class-specific covariates in both linear mixed sub-model and in the survival sub-model; Stamenic et al. [15] defined latent classes to assess the impact of serum creatinine on graft failure risk with no covariates in JLCM, and performed a multivariable multinomial logistic analysis after defining these latent classes in order to analyze the factors associated to the classes.

A few simulation studies concerning the JLCM and its extensions (competing risks, interval censoring, multi-state survival sub-model) were carried out [6, 16–18]. However, these simulations focus on the model usability and aim at validating the estimation procedure rather than exploring the general properties of the model and its finite-sample properties.

Thus the usage of the model is heterogeneous and its properties in terms of sample size and censoring rate are not comprehensively studied.

In this context, the objective of this paper is to empirically, by a simulation study, explore the asymptotic properties of the JLCM model, namely, the impact of the censoring rate and of the number of individuals on bias and normality of parameter estimates as well as on the quality of latent class identification. A real data application will also be carried out. Within this application, the impact of covariates omission and inclusion in the model on estimations and class membership prediction will be investigated.

## Methods

### Joint latent class model

The joint latent class model is composed of three sub-models: a multinomial logistic regression defining the probability of belonging to a latent class, a mixed linear model for each latent class describing the evolution of the longitudinal marker, and a survival model accounting for the time-to-event for each class. The sub-models are detailed as follows.

- **The multinomial logistic regression** is defined by *π*_*i*_*g*, the probability of individual *i* to belong to a given latent class *g*, conditional on a covariate vector *X*_*i*_:
  1 $${} \pi_{ig}=P({ c_{i}}=g|\pmb{X}_{i})=\frac{\mathrm{e}^{\xi_{0g}+{\pmb{X}_{i}^{T}}\pmb{\xi}_{1g}}}{\sum_{l=1}^{{G}}\mathrm{e}^{\xi_{0l}+{\pmb{X}_{i}^{T}}\pmb{\xi}_{1l}}},  $$
  where *c*_*i*_ is the latent class for patient *i*, $c_{i} \in (1, \cdots, G), \pmb {X}_{i}^{T}$ is a vector of explanatory variables for *i* necessarily independent of time, *ξ*_1*g*_ the vector of coefficients associated to the covariates effects within class *g*. Note that *ξ*_0*G*_=0 and *ξ*_1*G*_=0 to assure the model identifiability. If no prior information about the latent class is available, it is possible to use the marginal probability of the class *g*, $ \frac {e^{\pmb {\xi }_{0g}}}{\sum _{l=1}^{G}e^{\pmb {\xi }_{0l}}}$ in Eq. (1).
- **The mixed linear model** for a trajectory of a longitudinal marker of an individual *i* over time points *t*_*ij*_,*Y*_*ij*_ in a latent class *g* is defined as:
  2 $${}Y_{ij}|(c_{i}=g)={\pmb{X}_{1ij}^{T}}\pmb{\gamma}+{\pmb{X}_{2ij}^{T}}\pmb{\beta}_{g}+{\pmb{Z}_{ij}^{T}}\pmb{b}_{ig}+\epsilon_{ij},  $$
  where $\pmb {X}_{1ij}^{T}$ is the vector of explanatory variables common to all latent classes and *γ* the corresponding vector of coefficients, $\pmb {X}_{2ij}^{T}$ is the vector of class-specific explanatory variables with *β*_*g*_ the corresponding vector of coefficients, and *Z*_*ij*_ is the vector of explanatory variables associated with the random effects $\pmb {b}_{ig} \sim \mathcal {N} (\pmb {\mu }_{g}, \pmb {B}_{g})$ (*μ*_*g*_ is a mean of random effects, *B*_*g*_ is a variance-covariance matrix of random effects, both of which can be common or specific to latent classes). Note that $\pmb {X}_{1ij}^{T}$ and $\pmb {X}_{2ij}^{T}$ have no variables in common.
- **The survival model** for an individual *i* over time is defined by its hazard function, *α*_*i*_(*t*), within each latent class as:
  3 $$ \alpha_{i} (t)|\left(c_{i}=g\right) = {\alpha_{0}}\left(t,\pmb{\zeta}_{g}\right)\exp\left(\pmb{X}_{1i}^{T}\pmb{\vartheta}+{\pmb{X}_{2i}^{T}}\pmb{\eta}_{g}\right)  $$
  with *α*_0_(·) the baseline risk function in latent class *g*, parametrized by vector $\pmb {\zeta }_{g}, \pmb {X}_{1i}^{T}$ is the vector of explanatory variables and *𝜗* the associated parameters common to all latent classes, $\pmb {X}_{2i}^{T}$ is the vector of class-specific explanatory variables and *η*_*g*_ the corresponding class-specific parameters of the model.
  We denote by *T*_*i*_ the observed time to a clinical event of interest for individual *i*. In the framework of JLCM, it is important to note that the measures of the longitudinal marker after *T*_*i*_, if there exist, are excluded from the observed data. Indeed, the objective is to describe the link between the risk of the event and the marker change over time preceding the event. The observed duration $T_{i}= \min (T^{\star }_{i},C_{i})$, where $T^{\star }_{i}$ corresponds to the real time-to-event (possibly not observed) and *C*_*i*_ corresponds to the right-censored duration. The survival function corresponding to the hazard of Eq. (3), is defined as:
  4 $${} S(t)=\exp \left(-\int_{0}^{t} \alpha(u) du\right)  $$

Note that the individual covariate vectors $\pmb {X}_{i}^{T}$ can be different in each of the three sub-models (Eqs. (1)-(3)), but have same notations for simplicity.

### Likelihood

The parameters of the model can be estimated by the maximum likelihood method. The log-likelihood of the model defined for *G* latent classes is defined by Commenges and Jacqmin-Gadda [19] as:

5 $$ \begin{aligned} L(\pmb{\theta}_{G})=\sum_{i=1}^{N}\log \Big(\sum_{g=1}^{G}{\pi_{ig}}{f_{\pmb{y}_{i}|c_{i}}(\pmb{Y_{i}}|c_{i}=g)} \\ {\alpha_{i}(T_{i}|c_{i}=g)^{\delta_{i}}S_{i}(T_{i}|c_{i}=g)}\Big), \end{aligned}  $$

where *π*_*ig*_ is the probability of belonging to class *g* (Eq. (1)), $\phantom {\dot {i}\!}{f_{\pmb {y}_{i}|c_{i}}(\pmb {Y_{i}}|c_{i}=g)}$ is the probability density function of the longitudinal marker data in class *g*, defined in Eq. (2), *α*_*i*_(*T*_*i*_|*c*_*i*_=*g*) is the hazard function defined in Eq. (3), *S*_*i*_(*T*_*i*_|*c*_*i*_=*g*) is the corresponding survival function. The event indicator *δ*_*i*_ for each individual is defined as:

6 $$ \delta_{i}=\left\{\begin{array}{ll} 1, & \text{if } T^{\star}_{i}<C_{i}.\\ 0, & \text{otherwise}. \end{array}\right.  $$

The model parameters are estimated using the maximum likelihood estimator (MLE); the log-likelihood function is maximized by Newton-Raphson-like algorithm [20].

The optimal number of latent classes, *G*, is defined following Tofighi and Enders [21] by the BIC (Bayesian information criterion): the number of classes corresponding to the minimum value of BIC is preferred. However, the choice of *G* is also based on the number of patients per class and the concordance between the *a posteriori* classification derived from the model and expert opinion.

### Class prediction and goodness-of-fit

Model goodness-of-fit can be assessed by a measure of class prediction accuracy. The class membership can be identified by computing the posterior probability of belonging to a class *g* for each subject, based on the estimated model parameters. This probability is conditional on the observed covariate vector, i.e. the longitudinal data *Y* and the event times *T*, and is defined in Eq. (7):

7 $${} \begin{aligned} {\pi_{ig}}^{Y,T}&= P(c_{i}=g|\pmb{Y_{i}},T_{i},{\delta_{i}};\hat{\pmb{\theta}}_{G})\\ &= \frac{\hat{\pi}_{ig}f_{\pmb{Yi}|c_{i}}(\pmb{Y_{i}}|c_{i}=g; \hat{\theta}_{G}) \alpha_{i}(T_{i}|c_{i}=g; \hat{\theta}_{G})^{\delta_{i}} S_{i} (T_{i} |c_{i}=g; \hat{\theta}_{G})} {\sum_{l=1}^{G} \hat{\pi}_{il}f_{\pmb{Yi}|c_{i}} (\pmb{Y_{i}}|c_{i}=l;\hat{\theta}_{G})\alpha_{i}(T_{i}|c_{i}=l; \hat{\theta}_{G})^{\delta_{i}} S_{i} (T_{i} |c_{i}=l; \hat{\theta}_{G})}. \end{aligned}  $$

The subject *i* is assigned to a class *g* corresponding to the maximum estimated *a posteriori* probability *π*_*ig*_.

Other approaches to goodness-of-fit can be employed, in particular those based on different types of residuals corresponding to different sub-models. These approaches will not be developed in the present paper.

## Results

### Simulation study

In the present study the properties of the JLCM are assessed by Monte-Carlo simulations. Simulations focus on the general model properties, on the model robustness to the number of individuals and the number of events, and on the quality of class separation.

The general framework for the simulation study is presented below.

#### Simulations design

The simulations are carried out for different settings in terms of the number of individuals *n*, *n*={100,500,1000,5000}, and in terms of the censoring rate *τ*,*τ*={0.05,0.10,0.15,0.25,0.50}, allowing to explore both possible asymptotic directions: the number of individuals and number of observed events [22]. The capacity of the model to distinguish between the latent classes is investigated by considering two different settings in terms of class separation: *high separation* (the classes are very different in terms of longitudinal marker evolution) and *low separation* (the classes are quite similar). The censoring mechanism was independent from the event process and no covariates were included in simulated models. Given the complex likelihood function, the optimisation algorithm may not always converge. That’s why for each setting in terms of *n*, *τ* and class separation, 120 datasets were generated to assure obtaining at least 100 results in each setting. The distribution of each of the estimated parameters was then analyzed in terms of normality, relative bias and coverage rate. The normality was assessed graphically by quantile-quantile plots. Indeed, normality tests would often reject the null hypothesis due to outliers in parameter estimations (this situation is probable due to the likelihood complexity; it results in local maxima, but is rare in practice) and/or to high test power. The relative bias for a parameter *θ* is calculated as:

$$\begin{array}{@{}rcl@{}} RB(\theta,n) = \Bigg|\frac{\frac{1}{K}\sum_{h=1}^{K}\hat{\theta}_{n,h}- \theta}{\theta}\Bigg|, \end{array} $$

with $\frac {1}{K}\sum _{h=1}^{K}\hat {\theta }_{n,h}$ the average parameter estimation from the sample of *n* individuals over *K* Monte-Carlo runs, and *θ* the real parameter value. The absolute value will be considered.

The coverage rate was calculated for each model parameter as the percentage of coverage of the real value by the estimated confidence interval.

The capacity of the model to distinguish the latent classes is assessed by the percentage of correctly predicted class memberships.

#### Data generation

The real parameters were chosen to mimic the real data, described in Stamenic et al. paper [15], dealing with a prognostic tool for individualized prediction of graft failure risk within ten years after kidney transplantation, using serum creatinine progression as a longitudinal marker. Following Eqs. (1 - 4), the generated data were governed by the following general model:

$${}\left\{ \begin{array}{ll} \pi_{i1} =Constant & \\  \quad \quad \text{for a 2-class model} \xi_{01} =\ln \left(\frac{\pi_{i1}}{1- \pi_{i1}} \right), \text{ see Eq.~(1)} \\ Y_{ij}|(c_{i}=g)= \beta_{0g} + \beta_{1g} t_{ij} + b_{ig}+\epsilon_{ig} \\  \quad \quad b_{ig} \sim \mathcal{N}\left(0, \sigma^{2}_{b,g}\right), \epsilon_{ig} \sim \mathcal{N}\left(0, \sigma^{2}_{\epsilon,g}\right) \\ S(t)|(c_{i}=g) = \exp\left(-\left(\frac{t}{\zeta_{1g}}\right)^{\zeta_{2g}}\right) \\  \quad \quad T^{\star}\sim \mathcal{W}eibull \left(\zeta_{1g}, \zeta_{2g}\right) \\ M(t)|(c_{i}=g) = \exp\left(-\left(\frac{t}{\tilde{\zeta}_{1g}}\right)^{\tilde{\zeta}_{2g}}\right) \\  \quad \quad C\sim \mathcal{W}eibull \left(\tilde{\zeta}_{1g}, \tilde{\zeta}_{2g}\right), \\ \end{array} \right.$$

*M*(*t*) being the survival function of the censoring distribution and *C* the censoring time. Note that the fact that there is no covariate in logistic model for class membership implies constant probability for each class membership. The considered longitudinal model is a random intercept mixed model and it implies that in Eq. (2), $\pmb {X}_{1ij}^{T}$ is a zero matrix (no common covariates) and $\pmb {X}_{2ij}^{T} = \left (1 \quad t_{ij}\right)$. The considered survival and censoring distributions imply that the survival and censoring times are Weibull random variables. The parameters of the censoring distribution were chosen empirically to meet the required censoring rate given the corresponding survival distribution. These nuisance parameters are not presented in the article.

The time points for repeated measures of the longitudinal marker are fixed to 1, 3, 6, 12, 18 and 24 months, following Stamenic et al. [15]. The parameters vector for a 2-classes model, with class common random effect and error variance of mixed sub-model is as follows:

8 $$\begin{array}{@{}rcl@{}} \pmb{\theta} = \Big(\xi_{01}, \beta_{01}, \beta_{11}, \beta_{02}, \beta_{12}, \sigma^{2}_{b}, \sigma^{2}_{\epsilon}, \zeta_{11}, \zeta_{21}, \zeta_{12}, \zeta_{22} \Big). \end{array} $$

The real values for the parameters were chosen as follows:

1. *High separation* framework.
  This setting is directly derived from Stamenic et al. [15], resulted in *β*_01_=170,*β*_02_=100,*β*_11_=88 by year, *β*_12_=1.2 by year, $ \sigma ^{2}_{b,1}= \sigma ^{2}_{b,2}=50$ and $\sigma ^{2}_{\epsilon,1}=\sigma ^{2}_{\epsilon,2}=60, \zeta _{11}=4.5, \zeta _{21}=2, \zeta _{12}=50, \zeta _{22}=1.01$.
2. *Low separation* framework.
  In this setting the values of the mixed model from *high separation* are divided by 2 to obtain quiet similar classes in terms of longitudinal marker evolution; survival model as well as random parameters were not modified, resulting in *β*_01_=135,*β*_02_=100,*β*_11_=44 by year, *β*_12_=1.2 by year.

**Fig. 1:**
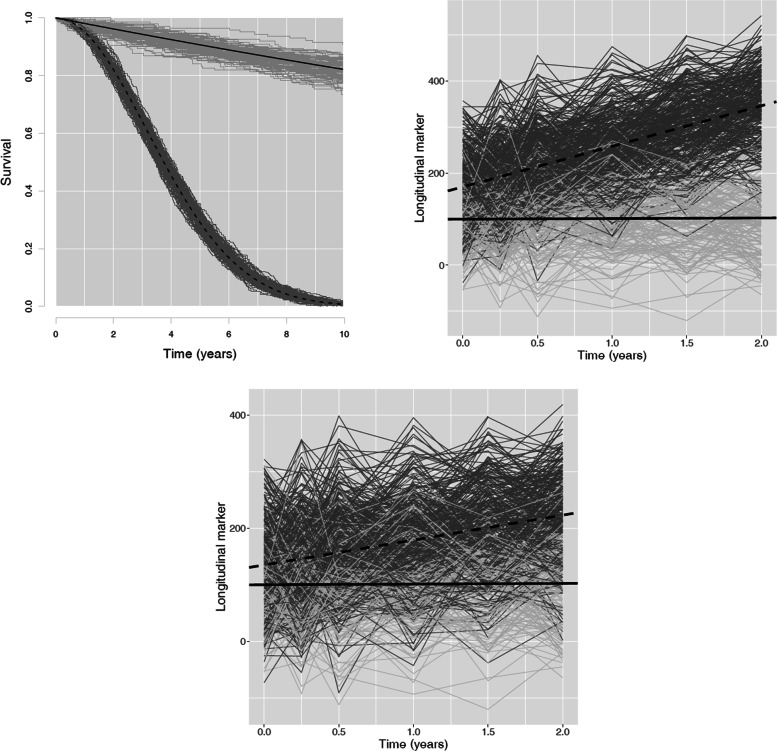
Simulated survival curves and longitudinal marker trajectories that mimic the real data from Stamenic et al. [15]. The number of individuals *n*=500; the censoring rate *τ*=0.05. Class 1: individual trajectories in darkgray, dashed line for mean trajectory; class 2: individual trajectories in lightgray, solid line for the mean trajectory. Figure at the top left: Generated survival curves for two classes and resulted examples of individual trajectories (same results for *high separation* and *low separation* settings). Figure on the top right: Simulated longitudinal marker evolution curves and the resulted examples of individual trajectories for the *high separation* setting. Bottom figure: Simulated longitudinal marker evolution curves and the resulted examples of individual trajectories for the *low separation* setting

In both settings, the shape parameter for the Weibull distribution for censoring was fixed to 1.5, inspired from real life, where more censoring occurs with time. The scale parameter for this distribution was empirically derived to meet the required censoring rate. The probability of class 1 membership was set to 0.3 in both settings, resulting in the logistic model parameter from Eq. (1) *ξ*_01_=−0.84. The examples of simulated trajectories for the *high separation* and *low separation* settings are illustrated in Fig. 1; the observed longitudinal trajectories are rather confounded in the *low separation* setting in comparison with the *high separation*.

#### Normality assessment

The normality of the estimated parameters is assessed by plotting quantile-quantile plots for each setting in terms of classes, the number of individuals *n* and of the censoring rate *τ*.

**Fig. 2:**
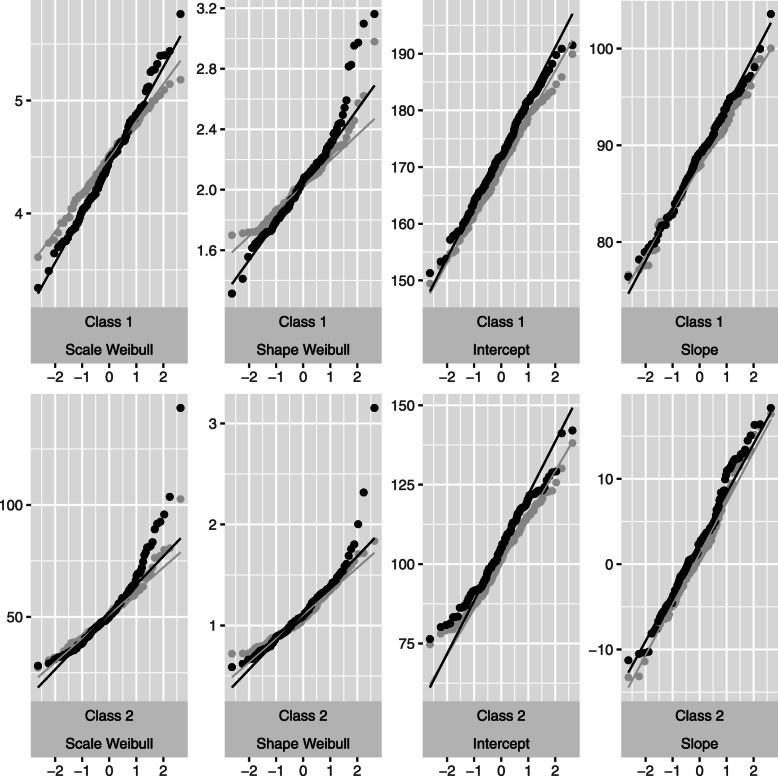
Simulations results : quantile-quantile plot for the parameter estimations, *high separation* setting, *n*=100. In black: censoring rate *τ*=0.5, in lightgray: *τ*=0.05. Results for Weibull scale, Weibull shape, mixed model intercept and mixed model slope, (*ζ*_1*g*_,*ζ*_2*g*_,*β*_0*g*_,*β*_1*g*_ respectively form Eq. (8)) are presented. *g*={1,2}

Figure 2 illustrates the results for the mixed and the survival sub-models, for 100 individuals, censoring rate 0.05 and 0.5 in the *high separation* setting. For small censoring rate (0.05) the normality of all the parameters is globally respected; heavy censoring (0.5) implies deviations from normality for the parameters of the survival sub-model.

Similar trends are observed for the other settings in terms of *n* and *τ* (results not presented). Note that the normality of the longitudinal sub-model parameters is not heavily impacted by small sample size and/or heavy censoring. Also, the MLE’s normality is not considerably influenced by the degree of class separation according to the present simulation study (results not presented). However, this conclusion should be considered with caution, since it can be different for different separation degrees.

**Fig. 3:**
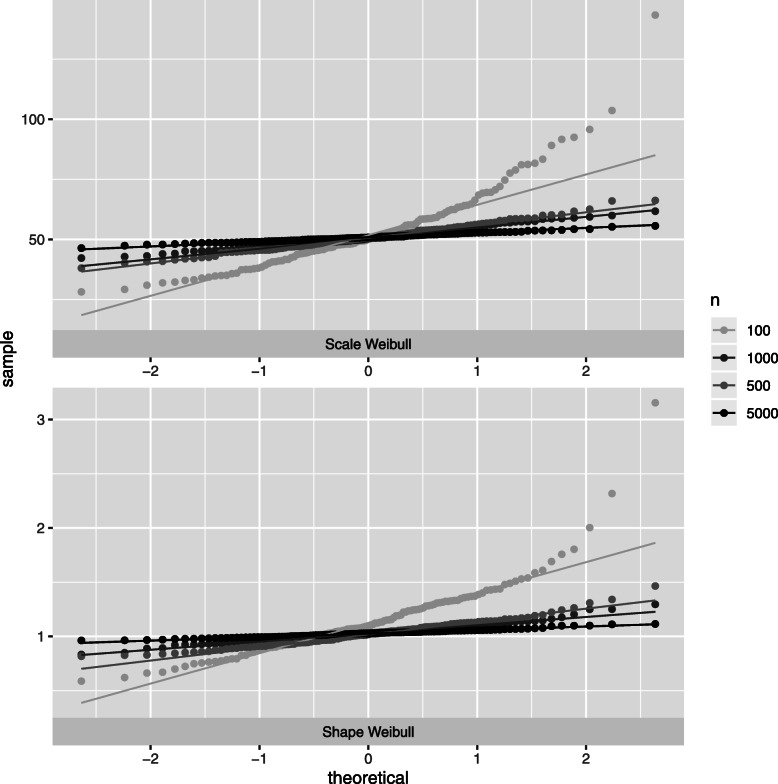
Simulations results : quantile-quantile plot for the parameter estimations, *high separation* setting, censoring rate *τ*=0.5. Results for Weibull scale and Weibull shape parameters of class 2 (*ζ*_12_ and *ζ*_22_ respectively form Eq. (8)) according to number of individuals *n* are presented

As expected, departures from normality decrease with increasing number of individuals (see Fig. 3 for the Weibull scale and shape parameters, heavy censoring) regardless of heavy censoring. Note that most of papers dealing with asymptotic properties of survival models are focused on the regression coefficients.Very few papers focus on the Weibull distribution parameters. Sirvanci and Yang [23] derives the asymptotic normality of the Weibull model parameters for Type I censoring data (fixed length of follow-up). However, in our study, empirically the departures from normality are reported for small sample size in terms of the number of events and/or the number of individuals (simulation results not presented here); in this sense, the normality problem is not specific to the joint latent class model, but is rather inherited from survival analysis.

#### Relative bias assessment

**Fig. 4:**
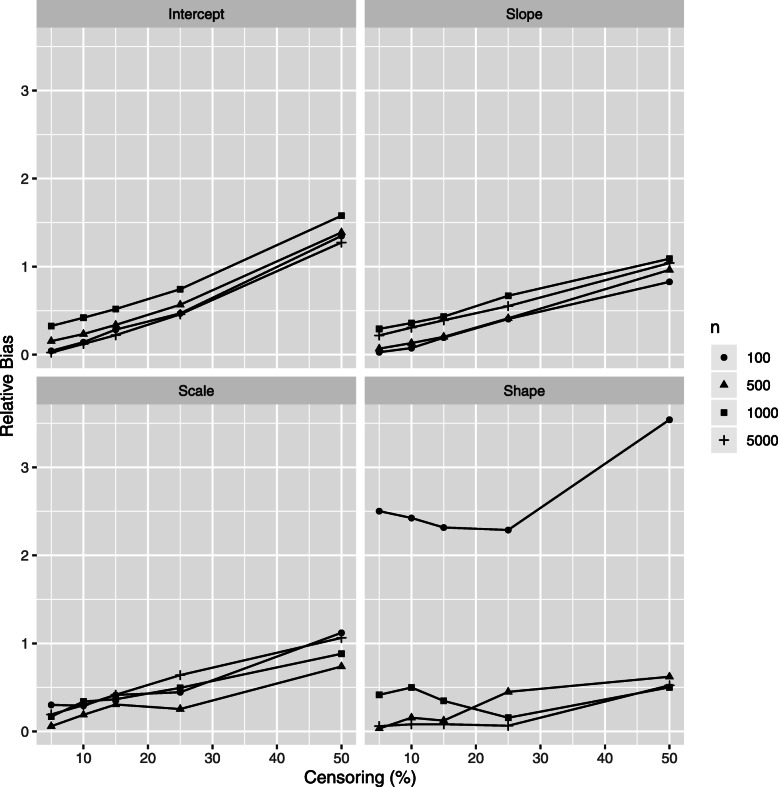
Simulations results: relative bias of the class 1 parameter estimations according to the censoring rate *τ* and to the number of individuals *n*, *high separation* setting. Results for mixed model intercept, mixed model slope, Weibull scale and Weibull shape, (*β*_01_,*β*_11_,*ζ*_11_,*ζ*_21_, respectively form Eq. (8)) are presented. Same vertical scale is used for the four figures

**Fig. 5:**
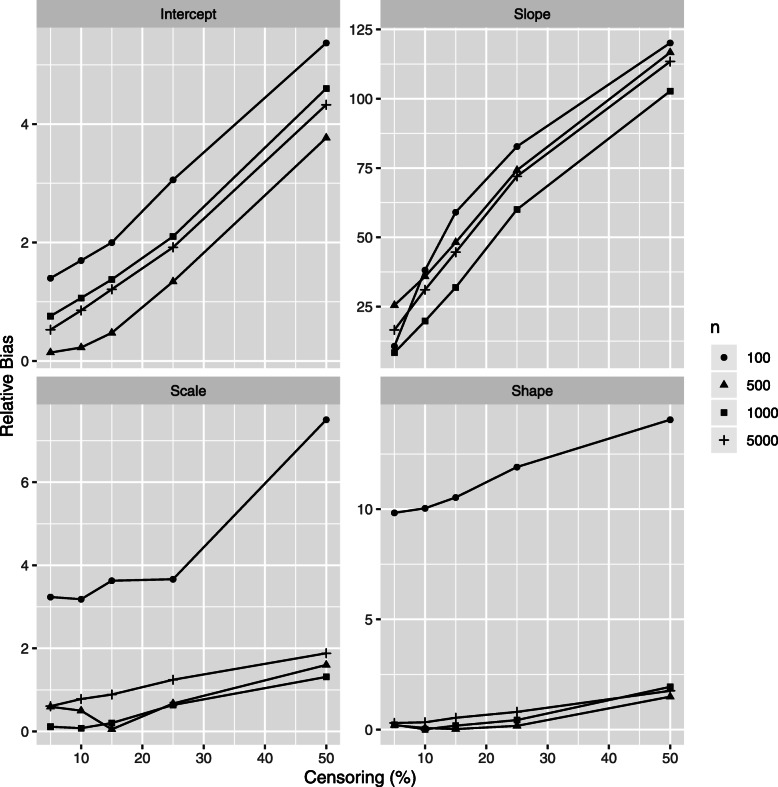
Simulations results: relative bias of the class 2 parameter estimations according to the censoring rate *τ* and to the number of individuals *n*, *high separation* setting. Results for mixed model intercept, mixed model slope, Weibull scale and Weibull shape, (*β*_02_,*β*_12_,*ζ*_12_,*ζ*_22_, respectively form Eq. (8)) are presented. A specific vertical scale is used for each figure

**Fig. 6:**
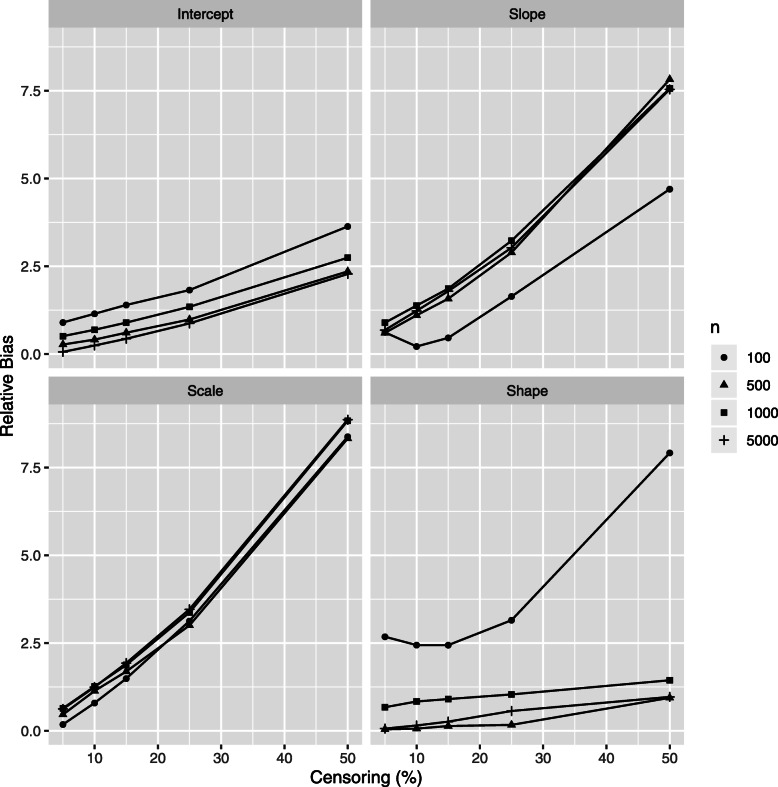
Simulations results: relative bias of the class 1 parameter estimations according to the censoring rate *τ* and to the number of individuals *n*, *low separation* setting. Results for mixed model intercept, mixed model slope, Weibull scale and Weibull shape, (*β*_01_,*β*_11_,*ζ*_11_,*ζ*_21_, respectively form Eq. (8)) are presented. Same vertical scale is used for the four figures

**Fig. 7:**
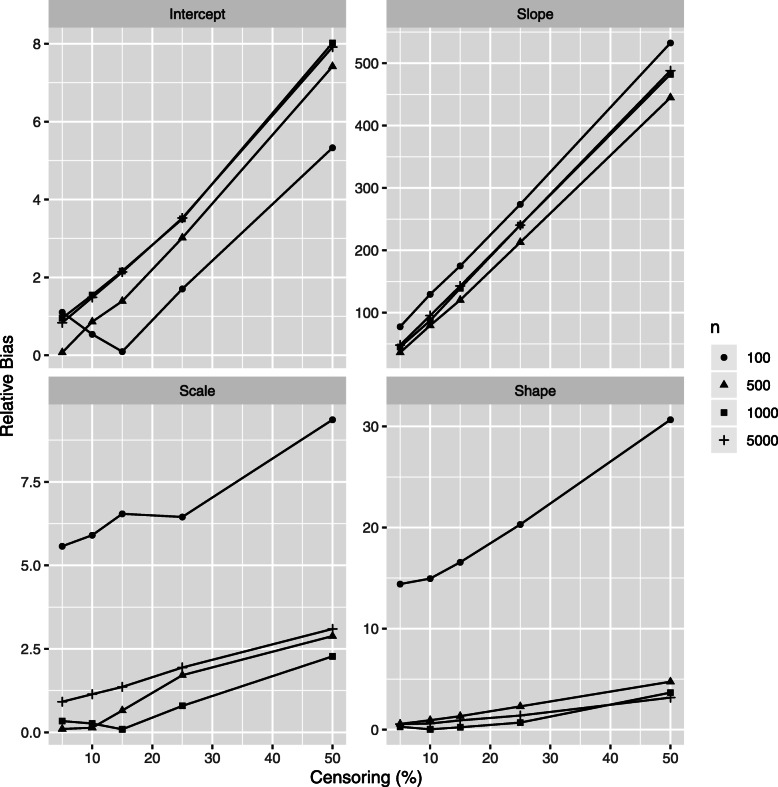
Simulations results: relative bias of the class 2 parameter estimations according to the censoring rate *τ* and to the number of individuals *n*, *low separation* setting. Results for mixed model intercept, mixed model slope, Weibull scale and Weibull shape, (*β*_02_,*β*_12_,*ζ*_12_,*ζ*_22_, respectively form Eq. (8)) are presented. A specific vertical scale is used for each figure

The relative bias (RB) of class-specific parameters estimates is illustrated in Figs. 4 and 5 for the *high separation* setting and in Figs. 6 and 7 for the *low separation* setting. The detailed numerical results are provided in Tables 1 and 2 for the *high* and *low separation* settings respectively.

**Table 1:** Simulations results: relative bias of model parameters for *high separation* setting according to the number of individuals, *n*, and to the censoring rate, *τ*

		Longitudinal sub-model						Survival sub-model			
*n*	*τ*	$\hat {\sigma }_{b} $	$\hat {\sigma }_{\epsilon } $	$\hat {\beta }_{0g}$		$\hat {\beta }_{1g}$		$\hat {\zeta }_{1g}$		$\hat {\zeta }_{2g}$	
				*g*=1	*g*=2	*g*=1	*g*=2	*g*=1	*g*=2	*g*=1	*g*=2
100	5	0.1053	21.1797	0.0429	1.3974	0.0281	10.7052	0.3018	3.2362	2.5023	9.8304
	10	0.0961	21.2395	0.1421	1.6954	0.0748	38.1642	0.2923	3.1830	2.4243	10.0367
	15	0.0997	21.3986	0.2846	1.9987	0.1936	59.0676	0.4148	3.6286	2.3160	10.5283
	25	0.0824	21.3810	0.4687	3.0567	0.4054	82.7586	0.4455	3.6646	2.2878	11.9084
	50	1.7125	21.8392	1.3486	5.3694	0.8271	120.1328	1.1201	7.5050	3.5401	14.0533
500	5	0.2267	21.4488	0.1534	0.1412	0.0685	25.4826	0.0591	0.5932	0.0332	0.2021
	10	0.2230	21.3820	0.2346	0.2261	0.1317	35.8174	0.1892	0.4996	0.1561	0.0727
	15	0.2062	21.4656	0.3380	0.4738	0.2027	48.1685	0.3062	0.0501	0.1232	0.0229
	25	0.2011	21.4412	0.5670	1.3390	0.4127	74.1988	0.2546	0.6707	0.4496	0.1703
	50	0.1945	21.6664	1.3876	3.7674	0.9627	116.6916	0.7377	1.6005	0.6217	1.4936
1000	5	0.0004	20.1935	0.3260	0.7563	0.2925	8.4122	0.1684	0.1125	0.4160	0.2180
	10	0.0015	20.2002	0.4197	1.0629	0.3599	19.7922	0.3399	0.0777	0.4994	0.0130
	15	0.0117	20.1928	0.5181	1.3744	0.4324	31.9018	0.3658	0.2015	0.3471	0.1781
	25	1.6848	20.2110	0.7436	2.1019	0.6690	60.0292	0.4954	0.6364	0.1559	0.4325
	50	0.0072	20.3934	1.5776	4.6004	1.0896	102.7405	0.8822	1.3124	0.5011	1.9393
5000	5	1.6342	19.9957	0.0242	0.5270	0.2185	16.5831	0.1929	0.6064	0.0605	0.2997
	10	1.6315	19.9945	0.1226	0.8526	0.3075	31.0770	0.2985	0.7797	0.0810	0.3320
	15	0.0380	19.9728	0.2215	1.2091	0.3905	44.6046	0.4162	0.8876	0.0823	0.5370
	25	0.0449	20.0112	0.4584	1.9183	0.5515	72.0024	0.6396	1.2441	0.0648	0.8037
	50	0.0400	20.2601	1.2738	4.3253	1.0406	113.4169	1.0629	1.8816	0.5238	1.7593

The estimations of the error and the random intercept standard deviations ($\hat {\sigma }_{\epsilon }$ and $\hat {\sigma }_{b} $ respectively), of the intercept and the slope ($\hat {\beta }_{0g}, \hat {\beta }_{1g}$ respectively) from the longitudinal sub-model and of Weibull scale and shape from the survival sub-model ($\hat {\zeta }_{1g}$ and $\hat {\zeta }_{2g}$ respectively) are presented. *g*: class membership identification

**Table 2:** Simulations results: relative bias of model parameters for *low separation* setting according to the number of individuals, *n*, and to the censoring rate, *τ*

		Longitudinal sub-model						Survival sub-model			
*n*	*τ*	$\hat {\sigma }_{b} $	$\hat {\sigma }_{\epsilon } $	$\hat {\beta }_{0g}$		$\hat {\beta }_{1g}$		$\hat {\zeta }_{1g}$		$\hat {\zeta }_{2g}$	
				*g*=1	*g*=2	*g*=1	*g*=2	*g*=1	*g*=2	*g*=1	*g*=2
100	5	0.2157	22.3405	0.9001	1.1061	0.6187	77.4912	0.1821	5.5733	2.6803	14.4064
	10	3.3939	22.1855	1.1485	0.5399	0.2169	129.4999	0.7945	5.9039	2.4429	14.9468
	15	0.2315	22.2708	1.3985	0.0946	0.4619	175.0008	1.4886	6.5439	2.4417	16.5623
	25	1.5321	22.1504	1.8233	1.7052	1.6413	273.7677	3.1293	6.4496	3.1511	20.3027
	50	1.3699	21.7546	3.6344	5.3290	4.6976	532.4839	8.3763	9.3616	7.9156	30.6630
500	5	0.2556	21.5565	0.2726	0.0707	0.6062	36.0688	0.4649	0.1000	0.0440	0.5662
	10	1.9185	21.3569	0.4126	0.8612	1.1048	79.5267	1.1380	0.1392	0.0640	0.9334
	15	3.4729	21.2975	0.6091	1.3911	1.5773	120.2866	1.6905	0.6533	0.1375	1.3348
	25	0.1728	20.9193	0.9847	3.0168	2.8914	212.8892	3.0037	1.7108	0.1707	2.2996
	50	1.7796	19.8756	2.3534	7.4185	7.8224	444.8984	8.3268	2.8825	0.9417	4.7449
1000	5	0.0184	20.1206	0.5111	0.9550	0.8944	44.8903	0.6416	0.3375	0.6727	0.2864
	10	0.0302	20.0392	0.6927	1.5469	1.3819	87.2876	1.2630	0.2644	0.8368	0.0338
	15	0.0424	19.8918	0.8955	2.1650	1.8632	139.1406	1.8765	0.0905	0.9058	0.2346
	25	0.0763	19.6487	1.3465	3.5027	3.2279	240.5047	3.3728	0.7920	1.0379	0.6998
	50	0.2146	18.4371	2.7442	8.0212	7.5645	482.3023	8.8312	2.2757	1.4414	3.6641
5000	5	1.6360	19.8545	0.0630	0.8358	0.6849	48.0499	0.6231	0.9167	0.0643	0.5431
	10	0.0529	19.7483	0.2447	1.4861	1.2312	95.3766	1.2469	1.1418	0.1531	0.6085
	15	1.5950	19.6407	0.4366	2.1381	1.7922	142.7663	1.9299	1.3603	0.2632	0.9193
	25	0.1003	19.3803	0.8741	3.5248	3.0292	240.2600	3.4624	1.9415	0.5656	1.3986
	50	0.2294	18.2465	2.2807	7.9142	7.5368	487.8892	8.8603	3.0965	0.9679	3.1725

The estimations of the error and the random intercept standard deviations ($\hat {\sigma }_{\epsilon }$ and $\hat {\sigma }_{b} $ respectively), of the intercept and the slope ($\hat {\beta }_{0g}, \hat {\beta }_{1g}$ respectively) from the longitudinal sub-model and of Weibull scale and shape from the survival sub-model ($\hat {\zeta }_{1g}$ and $\hat {\zeta }_{2g}$ respectively) are presented. *g*: class membership identification

The general trends for the RB range and for its evolution according to the sample size and to the censoring rate depend on model parameter and on degree of class separation. Concerning the variance parameters (the variance of error and of the random effect in the mixed sub-model) there is no clear trend in their RB evolution; the following trends are revealed for the remaining parameters:

- As for the **absolute values**, in the *high separation* setting (Fig. 6 and Table 1), the RB is the most important for two parameters of class 2: 1) the survival sub-model Weibull shape parameter (RB over 10% for small number of individuals) and 2) the mixed sub-model slope parameter (RB varies from 10% to 120% depending on number of individuals and on the censoring rate, the mean number of longitudinal markers in the worse case (100 patients and a censor of 50%) is 5.1). For the remaining parameters the RB does not exceed 10%. The trend is quite similar for the *low separation* setting (Fig. 6 and Table 2), but to a higher extent: the RB varies from over 30% to 530% in the worst setting (small *n* and high *τ*).
- As for the impact of the **censoring rate**, the RB increases linearly for a given number of individuals according to the decreasing number of events (increasing censoring rate). This trend is the same for both settings in terms of degree of class separation, but, in the same manner that the RB absolute values, in a higher extent for the *low separation* setting. Precisely, in the *high separation* setting the RB decreases by around 1% for the parameters of class 1 (2-8% in the *low separation* case) and for around 3-5% (2-15% in the *low separation* case) for the parameters of class 2, for the exception of the mixed model slope: 100% decrease in the RB in the *high separation* (respectively 400% in the *low separation* setting) for *τ* decreasing from 50% to 5%). Note that the linear trend for RB evolution in terms of *τ* is not always respected for small *n*.
- As for the impact of the **number of individuals**, the increasing *n* does not seem to strongly impact the RB. Moreover, the Weibull shape parameter is more influenced than the Weibull scale. Also, the *low separation* setting is more influenced than the *high separation* setting.

Note that class 2 has the least number of patients with a lower risk of death; therefore the parameters of this class are more affected by the censoring rate. Also, the high bias for the class 2 slope parameter is explained by the small theoretical value for this parameter (*β*_12_=1.2).

#### Coverage rate assessment

The coverage rate is globally satisfactory (refer to Tables 3 and 4 for the 95% coverage rates in the *high* and the *low separation* settings respectively). However, the large sample size in terms of the number of individuals results in smaller confidence intervals, entailing lower empirical coverage rate. This trend is especially visible for heavy censoring. Departures from normality already mentioned for these settings can also be a cause of this phenomenon.

**Table 3:** Simulations results: empirical coverage rates of estimated 95% confidence intervals according to number of individuals, *n*, and to the censoring rate, *τ*, *high separation* setting

*n*	*τ*	$\hat {\beta }_{0g}$		$\hat {\beta }_{1g}$		$\hat {\zeta }_{1g}$		$\hat {\zeta }_{2g}$	
		*g*=1	*g*=2	*g*=1	*g*=2	*g*=1	*g*=2	*g*=1	*g*=2
100	5	0.9664	0.9496	0.9328	0.9496	0.8824	0.8655	0.9580	0.9160
	10	0.9664	0.9496	0.9328	0.9412	0.8571	0.8655	0.9496	0.9076
	15	0.9664	0.9496	0.9412	0.9496	0.8824	0.8908	0.9412	0.9328
	25	0.9580	0.9496	0.9412	0.9580	0.8487	0.7899	0.9496	0.9076
	50	0.9328	0.9076	0.9748	0.9328	0.8403	0.7899	0.8824	0.8571
500	5	0.9667	0.9667	0.9833	0.9500	0.9250	0.9167	0.9333	0.9500
	10	0.9667	0.9750	0.9833	0.9417	0.9583	0.9250	0.9250	0.9500
	15	0.9667	0.9667	0.9750	0.9750	0.9250	0.9083	0.9417	0.9333
	25	0.9583	0.9500	0.9750	0.9417	0.8667	0.7750	0.9083	0.9167
	50	0.8750	0.9167	0.9333	0.9083	0.9000	0.8333	0.9333	0.9250
1000	5	0.9833	0.9333	0.9500	0.9250	0.8667	0.8750	0.9500	0.9417
	10	0.9750	0.9167	0.9500	0.9333	0.8833	0.9417	0.9583	0.9417
	15	0.9750	0.9167	0.9333	0.9333	0.8833	0.8917	0.9833	0.9583
	25	0.9500	0.9000	0.9167	0.9083	0.8917	0.8917	0.9417	0.9000
	50	0.8667	0.8000	0.8917	0.9083	0.9000	0.7000	0.9083	0.9167
5000	5	0.9750	0.9083	0.9333	0.8750	0.8917	0.9000	0.9667	0.9500
	10	0.9833	0.9083	0.9417	0.8583	0.9000	0.9250	0.9500	0.9417
	15	0.9667	0.8667	0.9083	0.8333	0.8250	0.8750	0.9417	0.9333
	25	0.9333	0.7917	0.9000	0.8250	0.8167	0.8667	0.8917	0.9333
	50	0.5000	0.2833	0.8000	0.7167	0.7750	0.7750	0.8500	0.9000

The results for the intercept and the slope from the longitudinal sub-model ($\hat {\beta }_{0g}, \hat {\beta }_{1g}$ respectively) and for the Weibull scale and shape from the survival sub-model ($\hat {\zeta }_{1g}$ and $\hat {\zeta }_{2g}$ respectively) are presented. *g*: class identification

**Table 4:** Simulations results: empirical coverage rates of estimated 95% confidence intervals according to number of individuals, *n*, and to the censoring rate, *τ*, *low separation* setting

*n*	*τ*	$\hat {\beta }_{0g}$		$\hat {\beta }_{1g}$		$\hat {\zeta }_{1g}$		$\hat {\zeta }_{2g}$	
		*g*=1	*g*=2	*g*=1	*g*=2	*g*=1	*g*=2	*g*=1	*g*=2
100	5	0.9664	0.9496	0.9328	0.9496	0.8824	0.8655	0.9580	0.9160
	10	0.9664	0.9496	0.9328	0.9412	0.8571	0.8655	0.9496	0.9076
	15	0.9664	0.9496	0.9412	0.9496	0.8824	0.8908	0.9412	0.9328
	25	0.9580	0.9496	0.9412	0.9580	0.8487	0.7899	0.9496	0.9076
	50	0.9328	0.9076	0.9748	0.9328	0.8403	0.7899	0.8824	0.8571
500	5	0.9667	0.9667	0.9833	0.9500	0.9250	0.9167	0.9333	0.9500
	10	0.9667	0.9750	0.9833	0.9417	0.9583	0.9250	0.9250	0.9500
	15	0.9667	0.9667	0.9750	0.9750	0.9250	0.9083	0.9417	0.9333
	25	0.9583	0.9500	0.9750	0.9417	0.8667	0.7750	0.9083	0.9167
	50	0.8750	0.9167	0.9333	0.9083	0.9000	0.8333	0.9333	0.9250
1000	5	0.9833	0.9333	0.9500	0.9250	0.8667	0.8750	0.9500	0.9417
	10	0.9750	0.9167	0.9500	0.9333	0.8833	0.9417	0.9583	0.9417
	15	0.9750	0.9167	0.9333	0.9333	0.8833	0.8917	0.9833	0.9583
	25	0.9500	0.9000	0.9167	0.9083	0.8917	0.8917	0.9417	0.9000
	50	0.8667	0.8000	0.8917	0.9083	0.9000	0.7000	0.9083	0.9167
5000	5	0.9750	0.9083	0.9333	0.8750	0.8917	0.9000	0.9667	0.9500
	10	0.9833	0.9083	0.9417	0.8583	0.9000	0.9250	0.9500	0.9417
	15	0.9667	0.8667	0.9083	0.8333	0.8250	0.8750	0.9417	0.9333
	25	0.9333	0.7917	0.9000	0.8250	0.8167	0.8667	0.8917	0.9333
	50	0.5000	0.2833	0.8000	0.7167	0.7750	0.7750	0.8500	0.9000

The results for the intercept and the slope from the longitudinal sub-model ($\hat {\beta }_{0g}, \hat {\beta }_{1g}$ respectively) and for the Weibull scale and shape from the survival sub-model ($\hat {\zeta }_{1g}$ and $\hat {\zeta }_{2g}$ respectively) are presented. *g*: class identification

#### Class membership prediction assessment

The quality of the class membership prediction is globally satisfactory (Table 5): it is over 90% for the majority of settings in terms of *n* and *τ*. However, this quality is globally weaker for the *low separation* setting (less than 95% comparing to a rate higher than 95% for the *high separation* setting) and for heavy censoring (83-85% for the *low separation* setting, censoring rate 0.5). A decreasing censoring rate results in a 1% to 3% of the class identification improvement for all *n*, for the exception of heavy censoring *τ*. The sample size *n* does not considerably influence the quality of predictions, and in the *low separation* setting the prediction accuracy is around 3-6% weaker compared to the *high separation* setting, for the exception of heavy censoring cases.

**Table 5:** Simulations results: class identification accuracy, calculated as the rate of correctly predicted class memberships, according to the number of individuals, *n*, and to the censoring rate, *τ*. The difference between the rates of the *high* and the *low separation* settings is provided

*n*	*τ*	*High separation*	*Low separation*	Difference
100	5	0.9760	0.9418	-0.0342
	10	0.9767	0.9347	-0.0420
	15	0.9748	0.9248	-0.0500
	25	0.9689	0.9039	-0.0650
	50	0.9556	0.8335	-0.1221
500	5	0.9790	0.9440	-0.0350
	10	0.9778	0.9376	-0.0402
	15	0.9764	0.9321	-0.0443
	25	0.9720	0.9148	-0.0572
	50	0.9586	0.8458	-0.1128
1000	5	0.9814	0.9477	-0.0337
	10	0.9798	0.9419	-0.0379
	15	0.9782	0.9354	-0.0428
	25	0.9745	0.9186	-0.0559
	50	0.9605	0.8488	-0.1017
5000	5	0.9817	0.9480	-0.0337
	10	0.9801	0.9417	-0.0384
	15	0.9784	0.9348	-0.0436
	25	0.9748	0.9189	-0.0559
	50	0.9618	0.8504	-0.1114

The obtained simulations results can be summarized as follows: in general the MLE properties of the model parameters are impacted by the number of individuals as well as by the number of observed events and the number of longitudinal observations, which are both governed by the censoring rate. Note that the frequency of longitudinal marker observations also determines the number of observed measures, although this parameter is left fixed in the present study.

The quality of class membership identification depends on the number of observed events rather than on the number of observed individuals. The degree of class separation, determined by the class-specific slope of the longitudinal model, influences the bias and the normality of the MLE as well as the class identification accuracy. The assessment of the model properties was carried out after removing simulations with estimation convergence problems. The convergence problems are principally due to initial parameter values used in numerical estimation procedure. Such situations are quite rare : 1/120 (0.8%) for the setting *n*=100 in *high separation* case and 9/120 times (7.5%) for the setting *n*=100 in *low separation* case. Other settings were not impacted.

### Real data application

In the present section, the analysis of the *Amyotrophic Lateral Sclerosis* (ALS) progression using a joint latent class model is presented.

ALS is a rapidly progressive and ultimately fatal neurodegenerative disease with an average life expectancy of 3–5 years from symptoms onset. However, longer than 10-years survival has been reported in 5–10% of patients [24, 25]. Despite numerous clinical trials dealing with treatments aimed at survival increase, only *riluzole* exhibited moderate efficacy [26]. One of the reasons which can explain the negative results of clinical trials is a strong heterogeneity of ALS patients in terms of the disease progression. The disease progression is generally measured at specific time points, resulting in a longitudinal marker. In this context, the joint latent class model, allowing to capture the patients heterogeneity and to simultaneously account for a longitudinal marker and a survival time, is better suited to analyze the ALS data.

The objective of our application is two-fold. Firstly, it is focused on capturing and describing the profiles of ALS patients in terms of the survival probability, the disease progression and clinical characteristics, described by covariates. Secondly, it aims at exploring the results in the light of model properties revealed by the simulation study.

#### Data collection

The data were collected in the framework of the *Trophos prospective cohort study* (TRO19622), a multicenter, randomized, placebo controlled, phase II/III clinical trial, which showed no efficacy of *olesoxime* in ALS [27]. The cohort consisted of 512 patients recruited across 15 European centres during the three-years period (2009–2011). The study time scale is the time since inclusion. The mean age of patients was 56 (*sd*=11.2) years at inclusion and 55 (*sd*=11.2) years at symptoms onset, with 331 (64.6%) men and 181 (35.4%) women. The diagnosis was definite in 107 patients (20.9%) and probable in 404 patients (79.1%) [28]; 101 (19.8%) patients suffered from bulbar form. The disease duration spanned between 6 and 36 months. Patients were treated with *50mgriluzole* twice a day for at least one month and had a baseline slow vital capacity (SVC) of 70%.

All patients were examined at inclusion and every 3 months thereafter for a maximum of 18 months for clinical, biochemical and hematological parameters. The disease-specific functional rating scale, revised ALSFRS (ALSFRS-R), was also assessed 1 month post-inclusion and then every 3 months until 18 months maximum. Survival time was defined as the duration between the date of disease onset and the date of a composite end-point: ALS-related death, tracheotomy, beginning of the non-invasive positive pressure ventilation (NIPPV) over 23 hours per day for 14 consecutive days or the date when last known to be alive.

#### Model construction

In terms of class identification, from 1 to 4 latent classes were considered. A quadratic trend for the longitudinal marker evolution was specified, and the corresponding mixed model was specific to each class, meaning that the quadratic terms were eliminated if not significantly different from 0, leading to a linear trend. The model performance in terms of class identification was assessed by the BIC.

To assess the impact of the sample size on parameter estimations, the estimations were carried out for the *whole sample* (512 patients) and for a subset of 100 randomly chosen patients. The results from the *reduced sample* appeared to be slightly different (results not presented here), reflecting the potential bias, revealed by the simulation study.

To better understanding of latent classes, modeling with and without covariates was performed. The covariates were included into the survival and the mixed sub-models, whereas the logistic regression, describing the probability of belonging to a class, was defined without covariates in all settings.

- **A model without covariates** (Eq. 9) includes a random-intercept mixed model with a class-specific quadratic function of time specified for the longitudinal marker evolution *Y*_*ij*_; the variances of the random effect ($\sigma ^{2}_{b}$) and of the error ($\sigma ^{2}_{\epsilon }$) were considered common to all classes. Survival curves are also considered as class-specific.
  The originally interval-censored survival times, collected at baseline and at months 1, 3, 6, 9, 12, 15 and 18, were imputed from a Weibull distribution of these interval-censored dates to obtain the exact event times. The imputation was carried out in order to obtain the setting close to that used in simulations. Specifically, a Weibull distribution was first fitted to the interval-censored dates, and then the exact event times were sampled from this distribution truncated by the limits of the observed intervals for each patient.
  9 $$ \left\{\begin{array}{ll} \pi_{ig}=\frac{\mathrm{e}^{\xi_{0g}}}{\sum_{l=1}^{{G}}\mathrm{e}^{\xi_{0l}}} \text{, from Eq.~(1)}\\ \\ Y_{ij}|(c_{i}=g)= \beta_{0g} + \beta_{1g} t_{ij} + \beta_{2g} t_{ij}^{2}+ b_{0i}+b_{1i} t_{ij}\\+b_{2i} t_{ij}^{2}+\epsilon_{i},& \\ \quad \quad \pmb{b}_{i} \sim \mathcal{N}\left(0, \pmb{B}\right), \epsilon_{ij} \sim \mathcal{N}\left(0, \sigma^{2}_{\epsilon}\right) \text{, from Eq.~(2)} \\ \\ S(t_{i})|(c_{i}=g) = \exp\left(-\left(\frac{t_{i}}{\zeta_{1g}}\right)^{\zeta_{2g}}\right),& \\ \quad \quad T^{\star}\sim \mathcal{W}eibull \left(\zeta_{1g}, \zeta_{2g}\right), \text{ from Eq.~(4)} \end{array} \right.  $$
  with *B* covariance matrix of random effects.
- **A model with covariates** (Eq. 10, the hazard function is specified for easier interpretation) was specified based on clinical expertise and a preliminary unpublished study. This model includes baseline individual characteristics in the random intercept mixed sub-model and in the survival sub-model; the impact of these characteristics is specified common to all classes, following the clinical considerations. The quadratic term of time for the mixed sub-model appeared to be not significantly different from 0 for this model and is thus removed. Baseline covariates and their interactions with time were as well chosen from clinical expertise.
  The following abbreviations are used: *AO* (Age at onset), *SO* (Symptom Onset), *BMI* (Body Mass Index), *MUSC* (Muscular capacity), *SVC* (Slow vital capacity), *MCV* (Mean corposcular volume).
  10 $$ {}\left\{ \begin{array}{lll} \pi_{ig}&=\frac{\mathrm{e}^{\xi_{0g}}}{\sum_{l=1}^{{G}}\mathrm{e}^{\xi_{0l}}} \text{, from Eq.~(1)}\\ \\ Y_{ij}|(c_{i}=g)&= \beta_{0g} + \beta_{1g}t_{ij} + \gamma_{1}SO_{i}+ \gamma_{2}BMI_{i} + &\\& \gamma_{3}MUSC_{i}+ \gamma_{4}SVC_{i}+ \gamma_{5}MCV_{i} + &\\& t_{ij} \times \left(\gamma_{6}SO_{i}+ \gamma_{7}MUSC_{i} + \gamma_{8}SVC_{i} \right)+ &\\& b_{0i}+b_{1i} t_{ij}+\epsilon_{ij}, &\\& b_{i} \sim \mathcal{N}\left(0, \sigma^{2}_{b}\right), &\\& \epsilon_{ij} \sim \mathcal{N}\left(0, \sigma^{2}_{\epsilon}\right) \text{from Eq.~(2)} \\ \\ \alpha_{i}(t)|(c_{i}=g) &=\underbrace{\zeta_{1g}^{\zeta_{2g}}\zeta_{2g} t^{\zeta_{2g} - 1}}_{\alpha_{0} (t)} \exp(\vartheta_{1}{SO}_{i} +\vartheta_{2}{BMI}_{i} + &\\& \vartheta_{3}{MUSC}_{i} + \vartheta_{4}SVC_{i} + &\\&\vartheta_{5}AO_{i}) \text{, from Eq.~(3)} \\ \end{array} \right.  $$

#### Real data analysis results

**Fig. 8:**
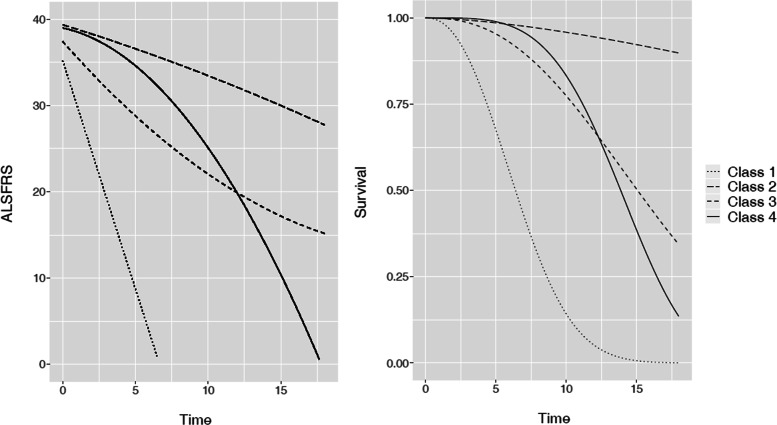
Real data results: class-specific estimations of the mean ALSFRS evolution and of the survival probability for the model without covariates

**Fig. 9:**
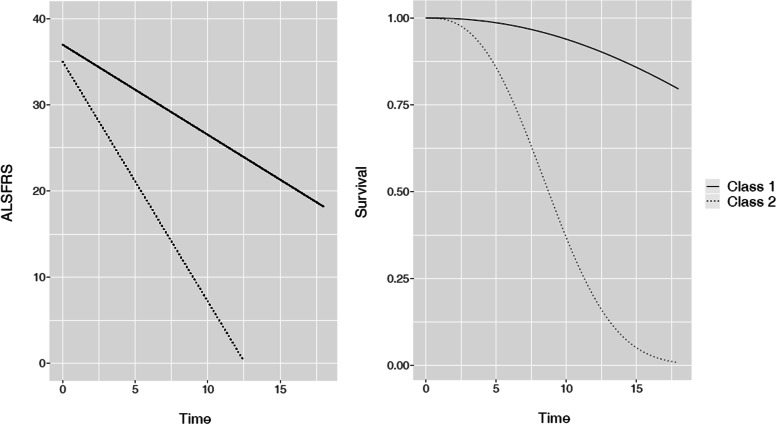
Real data results: class-specific estimations of the ALSFRS evolution and of the survival probability for a model with covariates. The mean values of explanatory variables were used for illustration

According to the BIC, 4 latent classes were retained for the model without covariates (BIC=15110 for 1 latent class, 14974 for 2 classes, 14911 for 3 latent classes and 14901 for 4 latent classes)and 2 latent classes for the model with covariates (BIC=14517 for 1 latent class, 14408 for 2 classes, 14410 for 3 latent classes and 14420 for 4 latent classes). Estimation results are presented in Table 6 and in Table 7 for the two models respectively. Models without and with covariates using the complete cases sample included 511 and 497 patients respectively. The difference in the number of patients is caused by missing covariates. Estimated survival curves and predicted ALSFRS evolution profiles are illustrated in Figs. 8 and 9 for the two considered models respectively.

**Table 6:** Real data results: parameter estimates with standard errors and *p*-values from the four-latent classes model **without covariates**

number of observations		2591	
number of patients		511	
average number of longitudinal measure		5	
number of events		132	
censoring rate		0.74	
Sub-model	Parameter	Estimate (se)	*p*-value
Multinomial logistic regression	*ξ*_01_	-0.29 (0.49)	0.55
	*ξ*_02_	2.26 (0.44)	<0.001
	*ξ*_03_	1.21 (0.53)	0.022
Weibull survival model	*ζ*_11_	0.37 (0.02)	<.001
	*ζ*_21_	1.52 (0.13)	<0.001
	*ζ*_12_	0.12 (0.02)	<0.001
	*ζ*_22_	1.25 (0.14)	<0.001
	*ζ*_13_	0.24 (0.01)	<0.001
	*ζ*_23_	1.56 (0.12)	<0.001
	*ζ*_14_	0.26 (0.01)	<0.001
	*ζ*_24_	2.02 (0.31)	<0.001
Linear mixed model : fixed effects	*β*_01_	35.22 (1.11)	<0.001
	*β*_11_	-5.29 (0.32)	<0.001
	*β*_21_	0.31 (0.04)	<0.001
	*β*_02_	39.37 (0.34)	<0.001
	*β*_12_	-0.52 (0.06)	<0.001
	*β*_22_	-0.01 (0.00)	0.007
	*β*_03_	37.44 (0.66)	<0.001
	*β*_13_	-1.92 (0.16)	<0.001
	*β*_23_	0.04 (0.01)	<0.001
	*β*_04_	39.00 (1.30)	<0.001
	*β*_14_	-0.36 (0.20)	<0.001
	*β*_24_	-0.10 (0.02)	<0.001
Linear mixed model : random effects	$\sigma ^{2}_{b_{0}}$	22.93	
	$\sigma ^{2}_{b_{1}}$	0.20	
	$\sigma ^{2}_{b_{2}}$	0.00	
	$\sigma ^{2}_{\epsilon,1}$	1.67

**Table 7:** Real data results: parameter estimates with standard errors and *p*-values from the two-latent classes model **with covariates**

number of observations		2525	
number of patients		497	
average number of longitudinal measure		5	
number of events		129	
censoring rate		0.74	
Sub-model	Parameter	Estimate (se)	*p*-value
Multinomial logistic regression	*ξ*_01_	2.22 (0.31)	<0.001
Weibull model	*ζ*_11_	0.48 (0.17)	0.004
	*ζ*_21_	1.48 (0.08)	<0.001
	*ζ*_12_	0.68 (0.21)	0.001
	*ζ*_22_	1.64 (0.12)	<0.001
	*𝜗*_1_	-0.05 (0.01)	0.008
	*𝜗*_2_	-0.05 (0.03)	0.079
	*𝜗*_3_	-0.03 (0.01)	<0.001
	*𝜗*_4_	-0.41 (0.12)	<0.001
	*𝜗*_5_	0.04 (0.01)	<0.001
Linear mixed model : fixed effects	$\hat {\beta }_{01}$	9.79 (4.02)	0.015
	*β*_11_	-2.32 (0.27)	<0.001
	*β*_02_	7.83 (4.12)	0.057
	*β*_12_	-4.06 (0.39)	<0.001
	*γ*_1_ (SO)	-0.06 (0.02)	<0.001
	*γ*_2_ (BMI)	-0.13 (0.05)	0.009
	*γ*_3_ (MUSC)	0.16 (0.00)	<0.001
	*γ*_4_ (SVC)	1.04 (0.18)	<0.001
	*γ*_5_ (MCV)	0.10 (0.04)	0.007 1
	*γ*_6_ (SO ×*t*_*j*_)	0.02 (0.00)	<0.001
	*γ*_7_ (MUSC ×*t*_*j*_)	0.01 (0.00)	<0.001
	*γ*_8_ (SVC ×*t*_*j*_)	0.06 (0.02)	0.018
Linear mixed model : random effects	$\sigma ^{2}_{b_{0}}$	14.10 (0.00)	
	$\sigma ^{2}_{b1}$	0.18	
	$\sigma ^{2}_{\epsilon,1}$	1.97	

Note: the following covariates and their interactions with time (if significant) are presented: SO (Symptom Onset), BMI (Body Mass Index), MUSC (Muscular capacity), SVC (Slow vital capacity), MCV (Mean corposcular volume)

The resulting latent classes are quite distinct both for the 4-classes no covariate model and for the 2-classes model including the covariates. The classes are characterized by a degree of ALSFRS decline and by the survival probability: a more rapid ALSFRS evolution is associated to a worse survival prognosis (refer to Figs. 8 and 9). In particular, the latent classes identified within the *model without covariates* can be interpreted in the following manner (refer to Fig. 8 for illustration).

- Classes 1 and 4 from the model without covariates are each composed of 5.1% of population. They represent patients with the most rapid decrease of ALSFRS and the highest risk of death, with a median survival around 7 months and 14 months for class 1 and 4 respectively.
- Class 2 is the largest (68.5% of patients) and is characterized by the slowest evolution of ALSFRS and the highest survival rate (median survival over 20 months).
- Class 3 is composed of 21.3% of population and represents an “average” class with an ALSFRS progression similar to that in class 1 but with a lower baseline value: from Table 6 we observe the baseline value of 37 in class 3 *vs* 39 for class 2. The survival probability in class 3 is lower than that in class 2, with a median survival around 15 months.

The latent classes identified within the *model with covariates* can be interpreted in the following manner (refer to Fig. 9 for illustration).

- Class 1 is the largest (92.6% of patients), is characterized by a moderate ALSFRS progression (-2.3 point by months) and by a better survival prognosis (over 20 months median survival compared to around 8 months for class 2, for a patient with the average covariates vector).
- Class 2 is composed only of 37 patients (7.4%) and describes a specific patient profile, worsening and dying very quickly.

Note that after adjustment on the pre-specified factors from literature, known to be associated to ALSFRS progression and survival, two latent patient profiles are identified by the model, indicating a lack of explanatory capacity of these factors and motivating the use of the latent class model. This remaining latency in the model with covariates confirms the interest of using the JLCM to analyze this kind of data, and suggests a need for further clinical analysis of the disease progression.

## Discussion

Several general considerations and recommendations concerning the use of the joint latent class model can be derived from the results of simulations.

To summarize, the departures from **normality** are particularly present for the survival sub-model parameters, and these departures disappear for a large enough number of observed events (small censoring rate) and/or large enough sample size (from 500 individuals normality is generally respected even for heavy censoring).

In terms of the **relative bias**, the trends are more complex. The parameters of the survival sub-model are also more impacted, especially for a small sample size *n*. The large number of individuals does not compensate for heavy censoring, as it was the case for normality. There is no particular trend in terms of *n*, except for the survival sub-model parameters, whose bias is considerably increased for *n*=100. The bias decreases quasi linearly for almost all parameters with increasing number of observed events (decreasing censoring rate). The estimations in the *low separation* case are less robust to the sample size and to the censoring rate than in the *high separation* case.

Finally, the **class identification accuracy** is slightly higher for the *high separation* setting and for smaller censoring, but not considerably influenced by the number of individuals, except for the case of heavy censoring; in the *low separation* setting the class identification accuracy is quite poor.

In the light of the obtained results, several remarks can be formulated concerning the general model usability.

Concerning implementation, the *low separation* setting, i.e., the small difference in the longitudinal model slopes, the likelihood optimization procedure is more likely to converge to a local maxima. Thus, several estimations with different initial parameter values should be carried out to assure that the obtained estimation is the global maxima.

Concerning the general model properties, the following should be accounted for.

1. Small sample size in terms of the number of individuals results in deviations from normality, especially for the survival model parameters. The provided confidence intervals may not be valid.
2. Heavy censoring implies bias in parameter estimation, especially in case of weak separation between latent classes. This bias is not compensated by large sample size.
3. Heavy censoring gives poor class identification accuracy, especially in the case of weak separation between latent classes.
4. The model parameters are generally more sensible to censoring rate than to the number of individuals in terms of bias, thus, increasing the time of observation is more beneficial for the accuracy of estimates than increasing the sample size in terms of the number of individuals.
5. In case of poor separation between latent classes, the bias increases and the class predictions accuracy decreases, the results should be interpreted with caution.
6. Small latent groups with few events (heavy censoring) should be characterized with caution, since the parameter estimations can be considerably biased.

As for the real data application results, using the joint latent class model for the described data is beneficial. Indeed, the latency remains in data after adjustment on covariates known from clinical expertise. Note however that the observed ALSFRS profiles are rather distinguished, i.e. the observed data are close to the *high separation* setting, implying better general results. As shown by simulations, in case of lower separation, it could be more difficult to obtain and interpret the latent classes. Moreover, the results obtained from the *whole* and *reduced* samples differ (results not presented). Thus, care should be taken when interpreting the parameters derived from small samples due to possible bias and inference problems resulting from departures from normality.

In the present paper, we focus on JLCM as the approach to account for unobserved heterogeneity when modelling censored longitudinal outcomes. Other alternatives to this approach exist as the mixed latent Markov models proposed by Bartolucci et al.

## Conclusion

The JLCM properties have been evaluated. We have illustrated the discovery in practice and highlights the usefulness of the joint models with latent classes in this kind of data even with pre-specified factors. We made some recommendations for the use of this model and for future research. Further work is needed to assess the role of covariates, their place in different sub-models of the JLCM, and the impact of their omission on parameter estimations and class membership identification. Also, precise recommendations concerning a minimum number of events or individuals needed to obtain satisfactory results within the JLCM can be formulated. Impact of longitudinal observation frequency on parameter estimations and latent classes identification can also be study considered in further work.

## Data Availability

The TROPHOS dataset analysed during the current study is publically available from https://pubmed.ncbi.nlm.nih.gov/27713255/

## Abbreviations

JLCM: Joint latent class model
MLE: Maximum likelihood estimations
GMM: growth mixture model
MMSE: Mini-mental state examination
ALS: amyotrophic lateral sclerosis
SVC: slow vital capacity
ALSFRS-r: amyotrophic lateral sclerosis functional rating scale revised, NIPPV non-invasive positive pressure ventilation
BIC: bayesian information criterion
